# The Risk Factors for Post-Bronchoscopy Respiratory Infection in Lung Cancer Patients—A Retrospective Case–Control Study from a Center in Greece

**DOI:** 10.3390/jcm14082848

**Published:** 2025-04-21

**Authors:** Vasileios Papavasileiou, Thomas Raptakis, Georgios Lavasidis, Georgios Niotis, Katerina Papavasileiou, Stefanos Lampadakis, Vasiliki Athanasopoulou, Pavlos Siozos, Kalliopi Theoni Vandorou, Garyfallia Nizami, Stelios Loukides, Ilektra Voulgareli

**Affiliations:** 1 2nd Respiratory Medicine Department, “Attikon” University General Hospital, 12461 Athens, Greece; tomraptakis76@yahoo.gr (T.R.); steflamp17@gmail.com (S.L.); vasiliki.athan95@gmail.com (V.A.); pavsioz@yahoo.gr (P.S.); kellyvandorou@gmail.com (K.T.V.); loukstel@med.uoa.gr (S.L.); ilektravoul@gmail.com (I.V.); 2 Evidence-Based Medicine Unit, Department of Hygiene and Epidemiology, University of Ioannina School of Medicine, University Campus, 45110 Ioannina, Greece; lavasidis@outlook.com.gr; 3 2nd Internal Medicine Department, “Attikon” University General Hospital, 12461 Athens, Greece; georgieni14@gmail.com; 4 IQVIA HELLAS A.E., 12461 Athens, Greece; katerinapapav1997@gmail.com; 5 Department of Ophthalmology, “ELPIS” General Hospital of Athens, 12461 Athens, Greece; filia_95@hotmail.com

**Keywords:** risk factors, bronchoscopy, respiratory infection, lung cancer

## Abstract

**Introduction:** Flexible bronchoscopy and its new methods have revolutionized the era of the diagnosis, staging, and restaging of lung cancer. A rare late complication is post-bronchoscopy respiratory infection, but it is critical due to treatment delays, treatment cancellation, and death. The aim of this study is to identify risk factors for respiratory tract infection after bronchoscopy in patients with lung cancer. **Methods:** A retrospective single-center observational study of 182 hospitalized patients was conducted at U.G.H. “ATTIKON” who underwent bronchoscopy for diagnosis/staging/restaging of lung cancer from January 2022 to April 2023. Patients were divided into two groups based on whether or not they developed post-bronchoscopy respiratory infection. **Results:** Analyzing the data between the groups, several potential risk factors for infection were identified, including recent hospitalization for COVID-19 within the last month (OR: 6.16; *p* = 0.01), history of COPD (OR: 8; *p* = 0.03), presence of emphysema on CT scan (OR: 8; *p* = 0.03), endobronchial lesions causing ≥ 50% bronchial obstruction with inability to advance the bronchoscope (OR: 9.6; *p* < 0.01), increased white blood cell count (≥8.5 K/μL) before bronchoscopy (OR: 8; *p* = 0.03), and advanced stage IV non-small-cell lung cancer (OR: 9.67; *p* = 0.02). **Conclusions:** Comparing our results with previous studies on risk factors for respiratory infections after bronchoscopy, we found that recent hospitalization for SARS-CoV-2 infection was a unique finding in our study. With the increasing incidence of lung cancer worldwide and the critical role of bronchoscopy in diagnosis/staging/restaging, large multicenter studies are needed to identify these risk factors and develop strategies for early detection, treatment, and prevention.

## 1. Introduction

Lung cancer is a heterogeneous disease in terms of both histological and molecular profiles, as categorized by WHO classifications. Since 2015, significant progress has been made in understanding the genetics of lung cancer and in developing molecularly targeted therapies [1,2,3].

As of 2024, lung cancer remains the second most common cancer and the leading cause of cancer-related death in both men and women [4]. Based on the results of a study in which 40 countries were included, the number of new cases of lung cancer for 2035 is expected to increase by 50% in both men and women compared with the number of cases in 2010. Therefore, early diagnosis and staging are critical [5].

Flexible bronchoscopy is the standard diagnostic procedure for patients with suspected lung cancer [6]. In addition to the diagnosis of lung cancer, it is used in its staging as well as in its restaging [7]. With the development of new techniques, such as methods in combination with endobronchial ultrasound (EBUS), the diagnostic range of bronchoscopy has been broadened [8,9,10].

Despite the safety provided as a test, this does not mean that there are no complications [7]. A rare late complication is a respiratory infection after bronchoscopy, which occurs in a low rate. This may be attributed to the fact that some clinical trials, such as AQuIRE, which is a multicenter study involving 15 centers in the United States and around 600 patients, include only complications occurring within 24 h [11]. Therefore, late complications, such as respiratory infections after bronchoscopy, would have been missed.

The incidence of respiratory infection after bronchoscopy ranges from 0.22% to 5.6% in the general population [10,12,13,14,15,16,17,18,19,20,21]. This type of infection is critical in patients with lung cancer. It not only causes delays in malignancy treatment but also cancer treatment cancellation and death [10]. In addition, pulmonary infections following bronchoscopy can deteriorate a patient’s functional status (Performance Status, PS), potentially necessitating alterations in anticancer therapy [13]. The incidence of this type of infection in lung cancer patients ranges from 3% to 6.3%, according to reports [13,14,17].

In this study, we sought to identify the risk factors of infectious complications after diagnostic bronchoscopy in patients with lung cancer.

## 2. Materials and Methods

### 2.1. Patients

This retrospective, single-center, patient-witness study was conducted in accordance with the Declaration of Helsinki and the guidelines and regulations of the Ethics Committee of University General Hospital (U.G.H.) “ATTIKON”, which approved the study and waived informed consent for retrospective data collection and specified analyses. Data from the medical records of patients who underwent diagnostic flexible bronchoscopy from January 2022 to April 2023 at the University General Hospital “ATTIKON” were collected retrospectively.

Inclusion criteria involved inpatients aged 18 years or older who underwent any type of diagnostic flexible bronchoscopy (simple or EBUS) resulting in cytological or histological confirmation of disease, as well as patients with a known history of primary lung cancer who underwent bronchoscopy for staging or restaging.

Patients with an already active respiratory infection at the time of bronchoscopy, patients without personal history of primary lung neoplasia who developed a respiratory infection after bronchoscopy, ICU inpatients, outpatients, and those whose medical records lacked information were excluded from the study.

Clinicopathological data were extracted retrospectively from the medical records of patients at our institution.

Among 184 patients with lung cancer who underwent diagnostic flexible bronchoscopy in the aforementioned time period, the data of 182 patients were analyzed due to the exclusion criteria from the study.

### 2.2. Data Collection

Data on symptoms, laboratory data, radiological data, bronchoscopic findings, histopathological results, and other relevant findings were collected.

Current smokers were defined as individuals who had smoked more than 100 cigarettes in their lifetime and had smoked in the last 12 months.

We defined a “central tumor” as a tumor involving <2 cm of the bronchial tree at or near the mediastinal or pericardial pleura and encompassing the trope, right and left main bronchus, and bronchial tree up to the segmental bronchus based on the multicenter study (RTOG) 0236.

Lung cancer staging was performed according to the 8th edition of the TNM classification.

Bronchoscopes (PENTAXEB19-J10, EBUS PENTAXEB19-J10U, Hamburg, Germany) were utilized, with the procedure performed by two or more experienced pulmonary specialists.

Oxygen saturation was continuously monitored by pulse oximetry, with target oxygen saturation being >90% during endoscopy. Lidocaine (2%) was applied topically to the upper airways prior to intoxication administration. For sedation during bronchoscopy, patients received intravenous midazolam with or without fentanyl. Each bronchoscopist decided which of the following procedures to use: bronchoalveolar lavage (BAL), bronchial brushing (brushing), endobronchial biopsy (EBB), conventional transbronchial biopsy (TBB), transbronchial needle aspiration using endobronchial ultrasound (EBUS-TBNA), transbronchial biopsy using endobronchial ultrasound (EBUS-TBNB), transesophageal fine-needle aspiration with ultrasound-guided bronchoscopic suction (EUS-B-FNA), or transesophageal bronchoscopic biopsy with ultrasound-guided needle aspiration (EUS-B-FNB). At least 5 biopsies were repeatedly obtained for pathological examination.

### 2.3. Definition of Lower Respiratory Tract Infection After Bronchoscopy

In this study, lower respiratory tract infection after bronchoscopy was defined as the combination of a compatible clinical presentation (prolonged febrile illness for >24 h after bronchoscopy, onset of dry cough or productive cough with purulent sputum, dyspnea) with laboratory deterioration (increased white blood cell count or leukopenia, increased CRP) and corresponding radiological confirmation, which led to the administration of antimicrobial therapy for at least 5 days. The period of post-bronchoscopy respiratory tract infection was defined as the interval starting 24 h after bronchoscopy and lasting up to 2 weeks [13].

### 2.4. Statistical Analysis

The mean describes quantitative variables, while absolute values and corresponding frequencies (%) represents continuous variables. The Mann–Whitney U test was used to compare the quantitative variables between the two groups, while the Fisher’s exact test was used for the qualitative variables. Statistical significance was defined as *p* < 0.05, and all analyses were conducted using R v2024.12.1+563.

## 3. Results

A total of 184 patients were diagnosed with primary lung cancer via bronchoscopy at U.G.H. “ATTIKON” between January 2022 and April 2023. Due to missing files for 2 patients, 182 patients were included in the study. During the study, 7 patients (3.9%) with a mean age of 67.1 years developed respiratory tract infections as a complication of diagnostic bronchoscopy (infection group), and 175 patients with a mean age of 71.7 years were included in the non-infection group. The demographic data of all patients included in the study are represented in Table 1.

Looking at the comorbidities of the two groups, no ultrasound-documented history of heart failure was found in the group of subjects who developed a respiratory infection, nor a history of diffuse interstitial lung disease (ILD), chronic kidney disease (CKD), cerebrovascular disease, malignancy, or autoimmunity. Conversely, patients who developed lower respiratory tract infections after bronchoscopy were predominantly those with Chronic Obstructive Pulmonary Disease (COPD) [n = 6 (85.7%) vs. n = 75 (42.9%), *p* = 0.046]. Also, another important element from the patients’ history was highlighted by recent hospitalization for SARS-CoV-2 in the last month before undergoing diagnostic bronchoscopy for histological documentation of lung cancer [n = 3 (42.9%) vs. n = 19 (10.9%), *p* = 0.04]. No significant difference in the Charlson comorbidity index was observed between the two groups [mean (range): 10 (8–13) vs. 8 (3–12)], although both groups exhibited elevated values. The location of the primary cancer focus and histological type did not reveal a significant statistical difference between the patients and controls. It is noteworthy that all patients in the infection group were stage IV for NSCLC or were extensive stage for SCLC. Stage IV of NSCLC indicated a statistically significant difference between the two groups [n = 6 (100%) vs. n = 67 (52.7%), *p* = 0.03]. Pre-bronchoscopy laboratory tests showed a higher frequency of elevated white blood cell counts (WBCs) in the infection group [mean (range): 14.82 K/μL (6.39–20.80) vs. 8.67 K/μL (3.67–14.60), *p* = 0.005], as seen in Table 2.

Chest CT findings revealed a higher prevalence of emphysema in the infection group [n = 6 (85.7%) vs. n = 75 (42.9%), *p* = 0.046]. In contrast, no significant statistical difference was found in other CT findings such as fibrosis, pleural effusion, or the presence of cavity/necrosis of the lesion [n = 2 (28.6%) vs. n = 11 (6.3%), *p* = 0.08].

Also, regarding the bronchoscopy itself, no statistically significant differences were found between the two groups regarding the duration of the bronchoscopy, the number of days after admission that the patient underwent this minimally invasive procedure, the type of anesthesia administered (midazolam with or without fentanyl), and the type of bronchoscopy he or she underwent (BAL, brushing, endobronchial biopsies, transbronchial biopsies, EBUS-TBNA/TBNB, EUS-B-FNA/FNB). The duration of bronchoscopy was significantly longer, in terms of minutes of examination [mean of patients 42 min versus controls 27.9 min, *p* = 0.07], but this was due to the fact that 42.9% of bronchoscopies were with endobronchial ultrasound (EBUS) in the patient group, whereas the corresponding percentage in the control group was only 22.9%, but no significant statistical difference was found here either (*p* = 0.38). Notably, sedation depth was not assessed using any standardized scoring system. It should also be noted that no patient from the infection development group underwent brushing. From the bronchoscopy findings, it was evident that the presence of endobronchial lesion with partial obstruction ≥ 50% and inability to further advance the bronchoscope was more common in the patient group than in the non-infection group [n = 2 (28.6%) vs. n = 7 (4%), *p* = 0.04]. No individual with complete bronchial obstruction from the lesion was found in the infection group. It is important to note that the bronchial generation (main, lobar, segmental, or sub-segmental) where endoscopic findings were observed was not recorded. The data of the CT findings and details on the bronchoscopy between the two groups are represented in Table 3.

Of the 7 patients (100%) in the infectious complication group, 5 (71.4%) developed pneumonia and 2 (28.6%) developed parapneumonic effusion, one of which was febrile. Three of the five patients who developed pneumonia had been hospitalized for COVID-19 infection in the last month. No lung abscess or mediastinitis was found. The time from bronchoscopy to symptom onset averaged 5 days, with a range of 1 to 12 days. A microbe was isolated in only one patient (14.3%) on sputum culture and blood culture, and this microbe was *Acinetobacter baumannii*. Of the 7 patients, 1 (14.3%) did not receive anticancer therapy. Two patients (28.6%) did not receive anticancer therapy due to poor functional status (Performance Status, PS), which was, however, pre-existing before the bronchoscopy was performed. No patient’s functional status changed after the bronchoscopy procedure. Finally, 4 patients (57.1%) received specific lung cancer treatment, with an average delay of 20 days (range: 13–26 days), compared with the control group.

## 4. Discussion

In this retrospective case–control study, we analyzed the clinical characteristics, imaging data, and bronchoscopic findings of patients who developed respiratory tract infections after diagnostic bronchoscopy, comparing them to a control group. Several potential risk factors for infection were identified, including recent hospitalization for COVID-19 within the past month (OR: 6.16, 95% CI: 1.28–29.63, *p* = 0.01), a history of COPD (OR: 8, 95% CI: 0.94–67.86, *p* = 0.03), presence of emphysema on CT (OR: 8, 95% CI: 0.94–67.86, *p* = 0.03), endobronchial lesions causing ≥ 50% bronchial obstruction with inability to advance the bronchoscope (OR: 9.6, 95% CI: 1.58–58.41, *p* < 0.01), elevated white blood cell count (≥8.5 K/μL) before bronchoscopy (OR: 8, 95% CI: 0.94–67.86, *p* = 0.03), and advanced stage IV non-small-cell lung cancer (OR: 9.67, 95% CI: 1.14–82.10, *p* = 0.02), as detailed in Table 4. When comparing our results with three prior studies on risk factors for respiratory infections after bronchoscopy, several of our findings were consistent with previously identified factors [13,14,17]. However, recent hospitalization for SARS-CoV-2 infection was a unique finding in our study.

The rate of respiratory infection complications in our study was 3.9%, aligning with rates reported in previous studies [13,14,17]. Although the exact mechanism of post-bronchoscopy infection remains unclear, it is hypothesized that bacteria from the oral cavity or upper airways may be introduced into the lower respiratory tract during the procedure [10,12,15,18,21,22,23,24]. The common pathogens associated with these infections include Streptococcus, Staphylococcus, Haemophilus influenzae, and anaerobic bacteria like Capnocytophaga species [13,17,23]. Despite strict bronchoscope sterilization protocols, bacterial contamination can still occur during the procedure. However, two other studies suggest that these complications may not be primarily related to oropharyngeal flora [10]. As the patient is hospitalized and, in most cases, receives antimicrobial treatment, the normal bacterial flora changes, and it is easy to colonize with a Gram-negative microorganism [25]. From our study, only one case of the pathogenic microbe was identified in both sputum culture and blood cultures, and this was an aerobic Gram-negative bacillus, Acinetobacter baumannii.

None of the three existing studies examined whether previous hospitalization for SARS-CoV-2 was a risk factor for this complication. A recent U.S. observational study indicated that while SARS-CoV-2 may cause less lung damage compared with other respiratory pathogens, it increases the risk of infection when hospitalization is necessary [26]. In our study, 42.9% of the patients who developed complications after bronchoscopy had been hospitalized for COVID-19 (Omicron variant) within the last month. Perhaps the development of subsequent infections may have been influenced by the fact that, according to international guidelines, many patients were taking immunosuppressive drugs such as dexamethasone at a dose of 6 mg/day for up to 10 days due to respiratory failure (67.5% of the period according to four centers in Greece, including “ATTIKON” hospital), while a smaller percentage (up to 4%) used immunomodulatory drugs such as baricitinib, anakinra, and others. Finally, it is worth noting that during the period of Omicron variant prevalence, hospitalized patients were typically older, had more comorbidities, and experienced more complications [27].

Although the pandemic from COVID-19 is several years behind, the virus continues to cause severe disease. According to data from 47 countries during the period 29 April to 26 May of 2024, there were 15,000 new admissions for COVID-19 disease [28]. As COVID-19 is still present, bronchoscopists should consider recent hospitalization due to this virus when taking patients’ medical histories.

Our study found that patients with emphysema on CT scan and a history of COPD had an increased risk of developing infectious complications after diagnostic bronchoscopy. In COPD patients, the lung mucosal surface is continuously exposed to microbial pathogens that have the potential to cause lower respiratory tract infection in susceptible hosts. The risk of developing pneumonia may be linked to host-related factors and changes in the microbiome, which can increase pathogen presence in the airways. Patients with COPD may be more prone to develop pneumonia based on their clinical features, such as chronic bronchitis with persistent mucus production. In particular, the presence of bacteria in the airway, both in stable COPD and during exacerbations, has been associated with increased inflammation and reduced host immune responses [29]. Perhaps the use of inhaled corticosteroids by some COPD patients may also play a role, as these medications are a known risk factor for pneumonia [30]. In our study, we did not examine whether the severity of COPD or the use of home-inhaled corticosteroids played a role in the development of this type of infection.

Smoking status (active smokers, ex-smokers, non-smokers) was not found to be a risk factor in our study, nor in those by Shimizu et al. and Shimoda et al. [13,17]. Smoking in patients with lung cancer causes mucosal inflammation and edema and compromises airway clearance from the mucosa [14]. Although smoking may contribute to pneumonia after bronchoscopy, its exact role remains unclear and requires further study.

Our laboratory data showed that elevated white blood cell counts in lung cancer patients predispose them to respiratory tract infections after endoscopy. This finding was also found in the study by Shimoda et al. [17]. Elevated white blood cell (WBC) counts have been shown to play a role in the immune and inflammatory processes of lung cancer pathogenesis [31].

For the above patients, for stage classification we used the eighth edition of TNM classification, which was implemented in the United States in January 2018. Staging is an essential cornerstone in the delivery of care for cancer patients. It provides a terminology to describe the tumor’s anatomical extent, as the characteristics of the tumor have a major impact on prognosis and treatment options. TNM is a useful tool to define prognosis, but multiple other factors influence prognosis [32]. In our study, all patients with respiratory tract infections were at an advanced stage of disease, a finding consistent with two other studies [13,17]. In addition, a study of W.-F. Tang et al. in 2022 showed that the distant metastases (stage IV in NSCLC) have been characterized by immunosuppression (low level of CD8+ T cells) and immune evasion (high level of PD-L1) [33]. But the involvement only of inpatients, who are generally older and have more comorbidities (with a mean Charlson comorbidity score of 10 in the infection group), may contribute to this finding. Also, the absence of developed screening guidelines for lung cancer patients in Greece [34] may play a role.

Finally, as in other studies involving lung cancer patients [13,14] or broader populations [16], the presence of an endobronchial finding as a risk factor for lung infection was emphasized. The percentage of stenosis caused by the endobronchial lesion was not found to be statistically significant in our study. It appeared that a lesion causing ≥ 50% stenosis with inability to further advance the bronchoscope may be a risk factor. The mechanism of pneumonia development after bronchoscopy in patients with tracheobronchial stenosis can be adequately explained in terms of that of post-obstructive pneumonia, as the presence of an endobronchial lesion affects airway clearance, predisposing patients to pulmonary infections [16,17].

The use of prophylactic antibiotics remains controversial among medical specialties. While the European Society of Gastrointestinal Endoscopy guidelines recommend prophylactic antibiotics, this is not supported by the American College of Chest Physicians (ACCP), the American Association of Bronchology, or the British guidelines for diagnostic flexible bronchoscopy in adults [12,17,18]. Many studies have administered antibiotics after bronchoscopy, failing to prevent respiratory tract infections [10,13,14]. However, Hayama et al. showed that prophylactic antimicrobials, started before the procedure and continued for the next three days, reduced the incidence of pneumonia after bronchoscopy to 4.2% in the prophylaxis group compared with 11.8% in the control group, although this difference was not statistically significant (*p* = 0.47) [35]. Prophylactic administration of beta-lactams did not have the desired statistical effect in Hayama’s study, perhaps due to the presence of microbes that do not belong to the antimicrobial spectrum of beta-lactams. Whether prophylactic antibiotic therapy is necessary for high-risk patients—and how to define these high-risk patients—needs further investigation through future multicenter studies. Given that endobronchial findings as a risk factor for infection are supported by two other studies in addition to ours, it remains to be determined whether starting antimicrobial therapy immediately after bronchoscopy could reduce this complication.

This study has limitations. It is a retrospective single-center study. The study involved only inpatients with lung cancer who underwent diagnostic bronchoscopy, and only a small number developed respiratory infections afterward. This relatively small number of patients who developed this rare complication is one of the disadvantages of the study. Data were obtained from the medical records of our hospital. Finally, as respiratory infections after bronchoscopy were identified retrospectively, they may have been underestimated due to strict diagnostic criteria (clinical, laboratory, and radiological confirmation). Thus, clinically mild cases such as exacerbations of COPD or acute bronchitis (which might not meet imaging criteria) may have been missed, though they likely did not affect subsequent lung cancer treatment.

## 5. Conclusions

This single-center retrospective case–control study highlighted the risk factors for developing respiratory infections in hospitalized lung cancer patients after the diagnostic bronchoscopy procedure. Identified risk factors included recent hospitalization for COVID-19, a history of COPD, presence of emphysema on CT scan, endobronchial lesions causing ≥ 50% bronchial obstruction with inability to advance the bronchoscope, elevated white blood cells (≥8.5 K/μL) before bronchoscopy, and stage IV NSCLC. Given the ongoing presence of COVID-19, bronchoscopists should consider recent hospitalization for this virus when taking a patient medical history. With the increasing incidence of lung cancer globally and the critical role of bronchoscopy in diagnosis, staging, and restaging, large multicenter studies are needed to identify these risk factors and develop strategies for early recognition, treatment, and prevention. The infrequent incidence of complications makes it difficult to conduct a randomized controlled trial. It is necessary to establish a uniform definition of the complication and then conduct multicenter studies to identify risk factors and establish guidelines for their prevention and early treatment.

## Figures and Tables

**Table 1:** Demographic characteristics of hospitalized patients included in the study.

Characteristics of Patients		Group with Respiratory Infection After Bronchoscopy n = 7 (3.9%)	Group Without Respiratory Infection After Bronchoscopy n = 175 (96.1%)	*p*-Value
Gender, n (%)	Female	3 (42.9%)	31 (17.7%)	0.12
	Male	4 (57.1%)	144 (82.3%)	
Age, mean (range), years		67.1 (55–83)	71.7 (53–86)	0.24
ΒΜΙ, mean (range), kg/m^2^		28.6 (21.9–35.2)	25.4 (19.6–34.6)	0.38
Smoking status, n (%)	Smokers	4 (57.1%)	111 (63.4%)	0.74
	Ex-smokers	2 (28.6%)	58 (33.1%)	
	Non-smokers	1 (14.3%)	6 (3.4%)	

**Table 2:** Personal history and laboratory data before bronchoscopy and characteristics of the tumors of patients included in the study.

Characteristics of Patients		Group with Respiratory Infection After Bronchoscopy n = 7 (3.9%)	Group Without Respiratory Infection After Bronchoscopy n = 175 (96.1%)	*p*-Value
Hospitalization for COVID-19 last month, n (%)		3 (42.9%)	19 (10.9%)	**0.04**
Comorbidities, n (%)	AH, n (%)	3 (42.9%)	98 (56%)	0.70
	DM, n (%)	2 (28.6%)	73 (41.7%)	0.70
	HF, n (%)	0 (0%)	45 (25.7%)	0.20
	Myocardial infarction, n (%)	1 (14.3%)	22 (12.6%)	1.00
	COPD, n (%)	6 (85.7%)	75 (42.9%)	**0.046**
	ILD, n (%)	0 (0%)	29 (16.6%)	0.60
	CRF, n (%)	2 (28.6%)	9 (9.7%)	0.06
	CKD, n (%)	0 (0%)	10 (5.7%)	1.00
	CVD, n (%)	0 (0%)	22 (12.6%)	1.00
	Malignancy, n (%)	0 (0%)	44 (25.1%)	0.20
	Autoimmune disease, n (%)	0 (0%)	15 (8.6%)	1.00
	Other, n (%)	1 (4.3%)	80 (45.7%)	0.13
CCI, mean (range)		10 (8–13)	8 (3–12)	0.18
Malignancy location, n (%)	RUL, n (%)	0 (0%)	75 (42.9%)	**0.04**
	RML, n (%)	0 (0%)	15 (8.6%)	1.00
	RLL, n (%)	0 (0%)	8 (4.6%)	1.00
	LUL, n (%)	4 (57.1%)	47 (26.9%)	0.10
	LLL, n (%)	3 (42.9%)	30 (17.1%)	0.11
	D ≥ 3 cm, n (%)	5 (71.4%)	152 (86.9%)	0.25
	Central location in CT, n (%)	5 (71.4%)	109 (62.3%)	1.00
Laboratory data prior to bronchoscopy	WBCs, mean (range), K/μL	14.82 (6.39–20.80)	8.67 (3.67–14.60)	**0.005**
	Alb, mean (range), g/dL	3.7 (2.9–4.3)	3.8 (2.7–4.7)	0.26
	CRP, mean (range), mg/L	76.2 (3.02–263)	21.3 (3.19–88.7)	0.11
Histological type of lung cancer	Adenocarcinoma, n (%)	3 (42.8%)	75 (42.9%)	1.00
	Squamous, n (%)	3 (42.9%)	52 (29.7%)	0.43
	SCLC, n (%)	1 (14.3%)	48 (27.4%)	0.68
Stage	Ι in NSCLC, n (%)	0 ° (0%)	0 * (0%)	1.00
	ΙΙ in NSCLC, n (%)	0 ° (0%)	25 * (19.7%)	0.59
	ΙΙΙ in NSCLC, n (%)	0 ° (0%)	35 * (27.6%)	0.34
	IV in NSCLC, n (%)	6 ° (100%)	67 * (52.7%)	**0.03**
	Limited in SCLC, n (%)	0 ^β^ (0%)	25 ^+^ (52.1%)	0.49
	Extensive in SCLC, n (%)	1 ^β^ (100%)	23 ^+^ (47.9%)	0.49

Bold: The *p*-value is smaller than 0.05. AH: Arterial Hypertension, DM: Diabetes Mellitus, HF: heart failure, COPD: Chronic Obstructive Pulmonary Disease, ILD: interstitial lung disease, CRF: chronic respiratory failure, CKD: chronic kidney disease, CVD: cerebrovascular disease, CCI: Charlson comorbidity index, RUL: right upper lung lobe, RML: right middle lung lobe, RLL: right lower lung lobe, LUL: left upper lung lobe, LLL: left lower lung lobe, D: diameter, WBCs: white blood cells; Alb: Albumin; CRP: C-reactive protein; SCLC: small-cell lung carcinoma; NSCLC: non-small-cell lung carcinoma; * is for NSCLC patients who have not developed a respiratory infection (127), ^+^ is for patients with SCLC who have not developed a respiratory infection (48), ° involves patients with NSCLC who have developed a respiratory infection (6), ^β^ involves a patient with SCLC who developed a respiratory infection (1).

**Table 3:** CT findings and details on the bronchoscopy of patients included in the study.

Characteristics of Patients		Group with Respiratory Infection After Bronchoscopy n = 7 (3.9%)	Group Without Respiratory Infection After Bronchoscopy n = 175 (96.1%)	*p*-Value
CT findings, n (%)	Emphysema, n (%)	6 (85.7%)	75 (42.9%)	**0.046**
	Fibrosis, n (%)	0 (0%)	29 (16.6%)	0.60
	Pleural effusion, n (%)	4 (57.1%)	53 (30.3%)	0.21
	Cavity/necrosis in the lesion, n (%)	2 (28.6%)	11 (6.3%)	0.08
Endobronchial finding, n (%)	Mucosal swelling, n (%)	1 (14.3%)	41 (23.4%)	1.00
	Infiltration of mucous membrane, n (%)	1 (14.3%)	93 (53.1%)	0.06
	Endobronchial lesion with partial obstruction < 50%, n (%)	1 (14.3%)	11 (6.3%)	0.38
	Endobronchial lesion with partial obstruction ≥ 50%, n (%)	2 (28.6%)	7 (4%)	**0.04**
	Endobronchial lesion with complete obstruction, n (%)	0 (0%)	15 (8.6%)	1.00
Bronchoscopy, n (%)	Duration of bronchoscopy, mean (range), minutes	42 (15–70)	27.9 (10–90)	0.07
	Midazolam, n (%)	7 (100%)	175 (100%)	1.00
	Fentanyl, n (%)	3 (42.9%)	135 (77.1%)	0.06
	Bronchial aspirations, n (%)	7 (100%)	175 (100%)	1.00
	BAL, n (%)	1 (14.3%)	10 (5.7%)	0.36
	Brushing, n (%)	0 (0%)	65 (37.1%)	**0.05**
	Endobronchial biopsies, n (%)	6 (85.7%)	115 (65.7%)	0.43
	Transbronchial biopsies, n (%)	0 (0%)	10 (22.9%)	0.35
	EBUS TBNA/TBNB, n (%)	3 (42.9%)	40 (22.9%)	0.36
	EUS-B-FNA/FNB, n (%)	1 (14.3%)	15 (8.5%)	0.48

Bold: The *p*-value is smaller than 0.05. CT: Computed Tomography scan; BAL: bronchoalveolar lavage; Brushing: cytological material collection using a brush; EBUS TBNA/TBNB: endobronchial ultrasound-guided transbronchial needle aspiration/biopsy; EUS-B-FNA/FNB: endoscopic ultrasound using bronchoscope fine needle aspiration/biopsy.

**Table 4:** The odds ratios of different variables for the diagnosis of respiratory infection after bronchoscopy.

	Odds Ratio	95% CI Lower Limit	95% CI Upper Limit	*p*-Value
Patient’s recent hospitalization (in the last month) for COVID-19 infection	6.16	1.28	29.63	**0.01**
Individual history of COPD	8.95	0.94	67.86	**0.03**
Presence of emphysema on CT scan	8.95	0.94	67.86	**0.03**
Presence of endobronchial lesion at bronchoscopy causing partial obstruction ≥ 50% of the bronchus with further inability to advance the bronchoscope	9.6	1.58	58.41	**<0.01**
White blood cells ≥ 8.5 K/μL in the patient’s blood before bronchoscopy	8	0.94	67.86	**0.03**
Stage IV in NSCLC	9.67	1.14	82.11	**0.02**

Bold: The *p*-value is smaller than 0.05.

## Data Availability

The original contributions presented in this study are included in the article. Further inquiries can be directed to the corresponding author.
